# *In Silico* Expressed Sequence Tag Analysis in Identification of Probable Diabetic Genes as Virtual Therapeutic Targets

**DOI:** 10.1155/2013/704818

**Published:** 2013-02-11

**Authors:** Pabitra Mohan Behera, Deepak Kumar Behera, Aparajeya Panda, Anshuman Dixit, Payodhar Padhi

**Affiliations:** ^1^Centre of Biotechnology, Siksha O Anusandhan University, Bhubaneswar, Odisha 751030, India; ^2^Hi-Tech Research and Development Centre, Konark Institute of Science and Technology, Techno Park, Jatni, Bhubaneswar, Odisha 752050, India; ^3^Department of Translational Research and Technology Development, Institute of Life Sciences, Nalco Square, Bhubaneswar, Odisha 751023, India

## Abstract

The expressed sequence tags (ESTs) are major entities for gene discovery, molecular transcripts, and single nucleotide polymorphism (SNPs) analysis as well as functional annotation of putative gene products. In our quest for identification of novel diabetic genes as virtual targets for type II diabetes, we searched various publicly available databases and found 7 reported genes. The *in silico* EST analysis of these reported genes produced 6 consensus contigs which illustrated some good matches to a number of chromosomes of the human genome. Again the conceptual translation of these contigs produced 3 protein sequences. The functional and structural annotations of these proteins revealed some important features which may lead to the discovery of novel therapeutic targets for the treatment of diabetes.

## 1. Introduction

To understand the behavior and functionality of various biological processes, it is important to get a clear cut idea of genes and gene products involved, evident by the regulatory interactions of DNA, RNA, and proteins. Rapid advancement in technologies like microarray, sequencing, and spectrometry has contributed vast data for analysis and prediction in the light of genomics and proteomics.

Expressed sequence tags (ESTs) are short stretch of nucleotide sequences (200–800 bases) derived from the cDNA libraries. These are capable of identification of the full-length complimentary gene and mostly used for the identification of an expressed gene. The EST generation process involves sequencing of single segments either 5′ end or 3′ end of random clones from cDNA library of an organism. A single sequencing reaction and automation of DNA isolation, sequencing, and analysis can generate many ESTs at a time. Since their original description and involvement as primary resources in human gene discovery [1], ESTs grow exponentially in various public databases, which will continue till there is suitable funding for the sequencing projects. Although the original ESTs were of human origin, a large number of ESTs are also isolated from model organisms like *Caenorhabditis elegans*, *Drosophila*, rice, and *Arabidopsis*. Public databases like dbEST [2], TIGR Gene Indices [3], and UniGene [4–6] now contain ESTs from a number of organisms for research and analysis. In addition, several commercial establishments maintain some privately funded, in-house collections of ESTs which are available for research. At present, ESTs are widely used throughout the genomics, and molecular biology communities for gene discovery, complement genome annotation, mapping, polymorphism analysis, gene prediction, gene structure identification, and expression studies establish the viability of alternative transcripts and facilitate proteome analysis.

Diabetes is a metabolic disorder characterized by hyperglycemia, glucosuria, negative nitrogen balance, and sometimes ketonemia. The clinical symptoms associated with it are retinopathy, neuropathy, and peripheral vascular insufficiencies. Overweight populations with sedentary lifestyle are more prone to diabetes. A recent study reveals that it affects 150 million people and almost 300 million more will be diabetic by the year 2025 [7]. Out of the three major types of diabetes, the non-insulin-dependent (type II diabetes or NIDDM) accounts for 90–95% of the diagnosed cases of the disease. There is no single approach to treat this disease and usually a combination therapy is adopted from different approaches. The worldwide epidemic of type II diabetes led the development of new strategies for its treatment. The discovery of nuclear receptor peroxisome proliferator activated receptors (PPARs) heralded a new era in understanding the patho-physiology of insulin receptors and its related complications [8]. PPARs are known to be the receptor for the fibrate class of hypolipidemic agents, while PPAR agonists reduce hyperglycemia without increasing the amount of insulin secretion. Again few other validated targets are protein tyrosine phosphatase-1B (PTP1B) and glycogen synthase kinase-3 (GSK-3). PTP-1B is a cytosolic phosphatase with a single catalytic domain [9]. *In vitro*, it is a nonspecific PTP and phosphorylates a wide variety of substrates. *In vivo*, it is involved in down regulation of insulin signaling by dephosphorylation of specific phosphotyrosine residues on the insulin receptor. GSK-3 is a type of protein kinase, which mediates the phosphorylation of certain serine and threonine residues in particular cellular substrates. This phosphorylation mainly inhibits the target proteins as in the case of glycogenesis it inhibits glycogen synthase [10–12]. While a lot of research is focused on validated targets like PTP1B, PPARs, and GSKs, this paper intends identification of novel diabetic genes as virtual target(s). The approach is purely* in silico* and by analysis of ESTs available in public databases.

## 2. Materials and Methods

### 2.1. Materials

Databases like dbEST, TIGR Gene Indices, and UniGene are most useful resources containing raw and clusters of ESTs for many organisms. The dbEST is the largest repository of EST data maintained by NCBI. The TIGR Gene Indices of DFCI alphabetically list the ESTs of many organisms. NCBI's UniGene contains gene-oriented clusters of transcript sequences obtained by alignments between transcript sequences and genomic sequences originating from the same gene. The current information content of these three databases is represented in Table 1.

To initiate an *in silico* analysis, the UniGene database was searched for human diabetes gene clusters that reported seven gene entries whose mRNA and ESTs information are listed in Table 2.

The ESTs of all seven gene entries were downloaded and only those originating from pancreas and liver tissue were taken for analysis. Only the 5′ ESTs were considered as the ESTs generated from the 3′ end are most error prone because of the low base-call quality at the start of sequence reads. There were no ESTs of pancreatic or hepatic tissue origin for the gene entry “Aquaporin 2 (AQP2).” Thus we found a total of 34 ESTs from six reported gene entries as listed in Table 3.

### 2.2. Methods

#### 2.2.1. EST Pre-Processing

The EST sequences are often of low quality because they are automatically generated without verification and thus contain higher error rates. The ESTs are also contaminated by vector sequences during their synthesis because a part of the vector is also sequenced along with the EST sequences. These sequences should be removed from EST to reduce the overall redundancy and to improve efficacy in further analysis. A comparison of ESTs with various nonredundant vector databases identifies the contamination which is deleted prior to analysis, for example. The EMVEC [13, 14] database removes the vector contamination from the EST sequences using NCBI BLAST2 [15, 16]. Using the UniGene clusters in our analysis is obvious as each cluster is generated by combined information from dbEST, GenBank mRNA database, and electronically spliced genomic DNA. Further they are clustered and cleaned from contamination (either by bacterial vector sequences or by linker sequences).

#### 2.2.2. EST Clustering and Assembly

The purpose behind EST clustering is to collect overlapping ESTs from the same transcript of a single gene into a unique cluster to reduce redundancy. This is important because all the expressed data coming from a single gene are grouped into an index class which represents information of that particular gene. The clustering or assembly is mainly done by pairwise sequence similarity search between sequences and it consists of three major phases. In the first phase, poor regions of both 5′ and 3′ reads are identified and removed. Then the overlapping regions between the sequences are calculated and the false overlaps are removed after their identification. In the second phase, reads are joined to form contigs in decreasing order of overlap scores. Then, both forward-reverse constraints are used to make corrections to the resulting contigs. In the third phase, a multiple sequence alignment of reads is constructed and a consensus sequence along with a quality value for each base is computed for each contig. Base quality values are used in computation of overlaps and construction of multiple sequence alignments. The tissue-based ESTs from six reported genes were subjected to cluster analysis by the CAP3 Server [17]. The subjected ESTs along with their gene names and resulting contigs are listed in Table 4. 

#### 2.2.3. Database Similarity Searches

The consensus sequences or contigs (putative genes) obtained from clustering are only useful if their functionality are ascertained and it is only possible by database similarity search using some freely available tools like BLASTN and BLASTX. For transcriptome analysis, the ESTs are additionally aligned to the genome sequence of the organism using specialized programs like BLAT (BLAST like alignment tool) [18] to assist genome mapping and gene discovery. The 6 contigs generated from 4 genes (GCK, AVPR2, ICA1, and SOX13) were subjected to BLAT analysis with parameters reading (genome: human, assembly: Feb. 2009 (GRCh37/hg19), query type: translated DNA, sort output: Score, output type: hyperlink). The outputs are listed in Table 5.

#### 2.2.4. Conceptual Translation of ESTs

**Figure 1:**
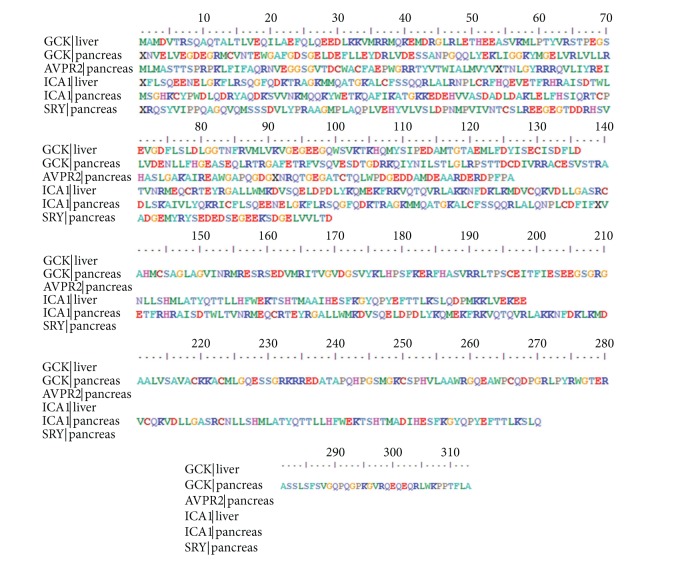
Graphical representation of protein sequences obtained from ESTScan2 translations and edited in BioEdit.

The EST sequences or data is informative only when its ontology, structure, and functions are obvious, for this the ESTs are correlated to protein-centric annotations by most accurate and robust polypeptide translations. The fact governing this process is that the polypeptides act as better templates for the identification of domains and motifs to study protein localization and assignment of gene ontology. The translations of ESTs are initiated by identifying the protein-coding regions or ORFs (open reading frames) from the consensus sequences or contigs. Here all 6 reported contigs were threaded to ESTScan2 [19, 20] tool with parameters reading (format: plain text, species: human, insertion/deletion penalty: −50, output: protein). The graphical view of 6 reported proteins is shown in Figure 1 obtained by BioEdit [21]. From these proteins, only 3 long continuous transcripts (GCK liver, GCK pancreas, and ICA1 liver) were selected for further structural and functional annotations.

#### 2.2.5. Functional Annotation

The functionality of a putative polypeptide is predicted by matching against nonredundant databases of protein sequences, motifs, and family; this is because proteins act as better templates for functional annotation implementing multiple-sequence alignment, profile, HMM generation, phylogenetic analysis, domains, and motif analysis. In our search for a novel diabetic gene, only 3 translated protein sequences (GCK liver, GCK pancreas, and ICA1 liver) obtained from ESTScan2 were subjected to InterProScan 4.8 [22] with parameters program: iprscan, nocrc: false, goterms: true, appl: blastprodom, fprintscan, hmmpir, hmmpfam, hmmsmart, hmmtigr, profilescan, hamap, patternscan, superfamily, signalp, tmhmm, hmmpanther, gene3d. The results are listed in Table 6.

## 3. Results and Discussion

Current EST analysis includes several steps and a wide range of computational tools are available for each step featuring different strengths and generate vital information systematically. Again there exists some arguments and confusion in selecting the suitable tools for individual steps of EST analysis and subsequent annotations at DNA and protein level. In our EST analysis for identification of novel diabetic genes as virtual targets for type II diabetes, we have followed a much cited procedure described by Nagaraj et al. [23]. From several successful and widely accessed EST databases, the UniGene database was selected as it uses mRNA and other coding sequence data of GenBank [24] as reference sequences for cluster generation. The UniGene clusters are updated weekly for progressive data management with the ever increasing EST data in GenBank. It stores all gene isoforms in a single cluster and does not generate consensus sequences. After search for the human diabetic gene in UniGene, the EST sequences of pancreatic and hepatic origin were selected due to their all-round association and greater functionality in the onset and continuation of diabetes. Only the 5′ ESTs of six genes were considered for analysis as the ESTs generated from the 3′ end are most error prone. After purposeful clustering of specific ESTs of a particular gene, we found out 1 contig each of hepatic and pancreatic origin for GCK, 1 contig of pancreatic origin for AVPR2, 1 contig each of hepatic and pancreatic origin for ICA1, and 1 contig of pancreatic origin for SOX13. The database similarity search by querying these contigs in BLAT against human genome revealed that both the hepatic contig and the pancreatic contig of GCK were showing good matches with chromosomes (1, 3, 4, 5, and 7) and (1, 3, 4, and X), respectively. The pancreatic contig of AVPR2 was showing good matches with chromosomes (1 and 2). Both the hepatic contig and pancreatic contig of ICA1 were showing good matches with chromosomes (1, 2, 5, and 7) and (1, 2, 7, and 16), respectively. The pancreatic contig of SOX13 was showing good matches with chromosomes (1, 6, and 7). The conceptual translation of these contigs in ESTScan2 provides six protein sequences from which we have considered only three (GCK liver, GCK pancreas, and ICA1 liver) as best for our analysis. The rest three sequences were left due to some erroneous readings (X, which does not code for any amino acid or refers to a stop codon) in their sequence. Thus the three proteins were GCK liver, a protein of 136 amino acids with molecular weight of 15474.87 Daltons; GCK pancreas, a protein of 313 amino acids with molecular weight of 34694.19 Daltons; and ICA1 pancreas, a protein of 270 amino acids with molecular weight of 31690.83 Daltons. These three proteins were named as hypothetical protein 1, hypothetical protein 2, and hypothetical protein 3 for further annotation.

### 3.1. The Hypothetical Protein 1

We have reported it from 5′ ESTs of liver tissues and it belongs to the hexokinase family of proteins with a distinct N-terminal and C-terminal. It is involved in the primary metabolic process like glycolysis and helps in the ATP-dependant conversion of aldohexose and ketohexose sugars to hexose-6-phosphate. The main function is carbohydrate kinase activity of various metabolic pathways like pentose phosphate pathway, fructose galactose metabolism, and glycolysis. It contains two structurally similar domains represented by PFAM families PF00349 [25] and PF03727 [26]. In structural classification by CATH, it belongs to the classification lineage of hierarchy 3.30.420.40 featuring 3 (alpha beta), 3.30 (2-layer sandwich), and 3.30.420 (nucleotidyltransferase; domain 5).

### 3.2. The Hypothetical Protein 2

We have reported it from 5′ ESTs of pancreas tissues and it also belongs to the hexokinase family of proteins with a distinct N-terminal and C-terminal. It is involved in the primary metabolic process like glycolysis and helps in the ATP-dependant conversion of aldohexose and ketohexose sugars to hexose-6-phosphate. The main function is the carbohydrate kinase activity of various metabolic pathways like pentose phosphate pathway, fructose galactose metabolism, and glycolysis. It contains two structurally similar domains represented by PFAM families PF03727 and PF00349. In structural classification by CATH, it belongs to the classification lineage of hierarchy 3.40.367.20 featuring 3 (alpha beta), 3.40 (3-layer (aba) sandwich), and 3.40.367 (hexokinase; domain 1).

Thus both hypothetical protein 1 and hypothetical protein 2 belong to the same family of proteins with common functions, but structurally they have different domains. The hexokinases contain 7 distinct motifs from which the motif 1 encodes the putative ATP-binding domain and motif 2 encodes for the sugar-binding domain. All motifs, except motif 6, contain amino acids that project into or near the ATP/sugar-binding pocket. Previously we have assumed that the glucokinase (GCK) gene is expressed and functions irrespective of the tissue types, but now it is obvious that there exit some structural differences although they function as same.

### 3.3. The Hypothetical Protein 3

We have reported it from 5′ ESTs of liver tissues and it belongs to a family of proteins containing an arfaptin domain with a distinct N-terminal and C-terminal. The arfaptin domain interacts with ARF1 (ADP-ribosylation factor 1), a small GTPase involved in vesicle budding at the Golgi complex and immature secretory granules. The structure of arfaptin shows that, upon binding to a small GTPase, arfaptin forms an elongated, crescent-shaped dimer of three-helix coiled coils [27]. The N-terminal region of ICA69 is similar to arfaptin [28]. It is involved in a neurological system process and secretes several neurotransmitters. It is also involved in a cellular process like cell communication and cell-cell signaling with synaptic transmission. In structural classification by CATH, it belongs to the classification lineage of hierarchy 1.20.1270.60 featuring 1 (mainly alpha), 1.20 (up-down bundle), 1.20.1270 (substrate binding domain of DNAk; chain A; domain 2), and 1.20.1270.60 (Arfaptin, Rac-binding fragment, chain A). Again we also found that the mammalian islet cell autoantigen (ICA69) is a 69 kDa protein [29].

### 3.4. Molecular Modeling of the Three Hypothetical Proteins

**Figure 2:**
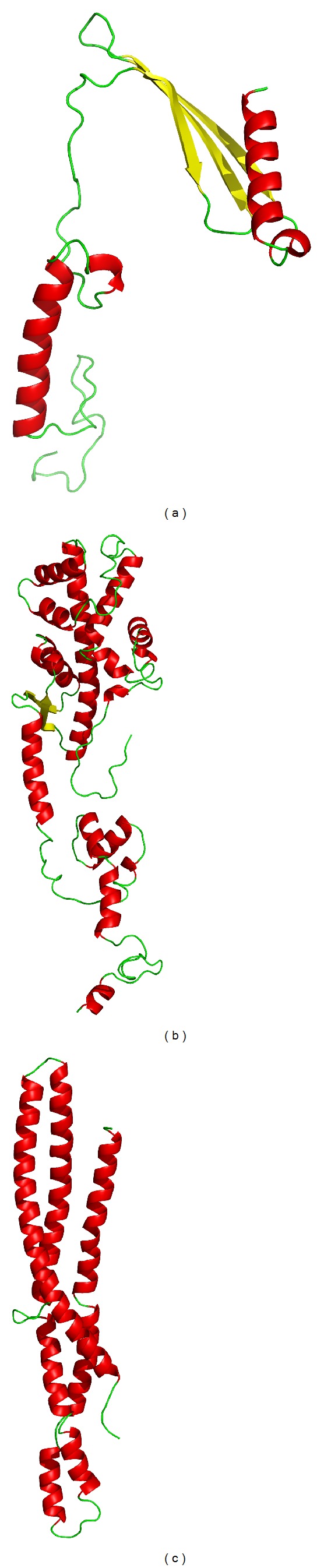
3D representation of homology models of three hypothetical proteins. (a) the homology model of hypothetical protein 1, (b) the homology model of hypothetical protein 2, and (c) the homology model of hypothetical protein 3.

To initiate the structural annotations of our three hypothetical proteins, we have generated the homology models using Modeller 9.10 [30–33]. The three protein sequences were queried in BLASTP [34] against the PDB [35] database to select their suitable templates. The template for hypothetical protein 1 was 1HKC with 50% sequence identity and a resolution of 2.80 Å. The template for hypothetical protein 2 was 2NZT with 48% sequence identity and a resolution of 2.45 Å. Similarly the template for the hypothetical protein 3 was 1I49 with 34% sequence identity and a resolution of 2.80 Å. After modeling, the three models were subjected to evaluation by development of the Ramachandran plots using PROCHECK NT stand-alone version [36]. The statistical information gathered from the Ramachandran plots revealed that 95.10%, 93.60%, and 95.70% of the residues were in the allowed region for the proteins hypothetical protein 1, hypothetical protein 2, and hypothetical protein 3 at an average resolution of 2.68 Å. Thus the models were perfect for structural annotation. The hypothetical protein 1 had 4 *α*-helices and 3 *β*-sheets arranged in a 2-layered sandwich model (Figure 2(a)). The hypothetical protein 2 had 16 *α*-helices and 2 *β*-sheets arranged in a 3-layer sandwich model (Figure 2(b)). The hypothetical protein 3 had only 7 *α*-helices acquiring an up-down bundle model (Figure 2(c)). Therefore the structural annotations by both InterProScan and subsequent modeling were just similar which purposefully validate our work.

In general glucokinase occurs in human liver, pancreas, gut, and brain cells and plays an important role in suitable regulation of carbohydrate metabolism. It works as a glucose sensor and triggers shifts in metabolism or cell function in response to the rising or falling levels of glucose. A mutation of the gene for this enzyme causes several forms of diabetes or hypoglycemia. Human islet cell autoantigen 1 protein is encoded by the ICA1 gene [37, 38]. This protein contains an arfaptin domain and is found in both cytosolic and membrane-bound Golgi complex and immature secretory granules. It also works as an autoantigen in insulin-dependent diabetes mellitus. Our *in silico* analysis revealed three new proteins from which two (hypothetical protein 1 and hypothetical protein 2) were functionally similar to glucokinase and one (hypothetical protein 3) was functionally similar to the human islet cell autoantigen 1. Due to their association in diabetes, these can be treated as virtual therapeutic targets for treatment of diabetes. There were structural variations among these three proteins and their functional homologues which need further structural analysis and interpretation.

## 4. Conclusion

The *in silico* EST analysis of seven reported genes associated with diabetes produced 6 consensus contigs which were annotated functionally and structurally. The functional annotations were similar to the corresponding proteins in which the ESTs were actually categorized. The structural annotations revealed that there is a variation which may be due the differences in source tissue types. This information can be used for further structure-based annotations, and new drug designs for the treatment of diabetes.

## Figures and Tables

**Table 1:** Information content for *Homo sapiens. *

Sl. no.	Database name	Release	Date	Information content
1	dbEST	040112	April 01, 2012	ESTs 8315296

2	TIGR Gene Indices	17.0	July 28, 2006	ESTs 7233257
				HTs 234976

3	UniGene	—	December 23, 2011	mRNAs 209412
				Models 212
				HTC 20115
				3′ ESTs 1693253
				5′ ESTs 4027153
				Unknown ESTs 927242
				Total sequences 6877387

**Table 2:** UniGene information on human diabetes (mRNA and ESTs).

Sl. no.	Name of the gene	Source	mRNA	ESTs
1	Glucokinase (GCK)	*Homo sapiens *	12	46
2	Arginine vasopressin receptor 2 (AVPR2)	*Homo sapiens *	14	10
3	Aquaporin 2 (AQP2)	*Homo sapiens *	07	61
4	Islet cell autoantigen 1 (ICA1)	*Homo sapiens *	10	217
5	SRY (sex determining region Y) box 13 (SOX13)	*Homo sapiens *	09	180
6	Ras-related associated with diabetes (RRAD)	*Homo sapiens *	06	160
7	Ankyrin repeat domain 23 (ANKRD23)	*Homo sapiens *	06	141

**Table 3:** ESTs reported from different genes.

Sl. no.	GB accession no.	Description	Tissue type	EST type	Code*
1	Glucokinase (GCK)				
	DA640823.1	Clone LIVER2005873	Liver	5′ read	P
	DA637293.1	Clone LIVER2000237	Liver	5′ read	P
	DA638310.1	Clone LIVER2002033	Liver	5′ read	P
	CK823298.1	Clone IMAGE:6136115	Pancreas	5′ read	P
	BM966889.1	Clone IMAGE:6136115	Pancreas	5′ read	P
	BM966913.1	Clone IMAGE:6135860	Pancreas	5′ read	P
	BQ101045.1	Clone IMAGE:6135541	Pancreas	5′ read	P

2	Arginine vasopressin receptor 2 (AVPR2)				
	BG830436.1	Clone IMAGE:4908956	Pancreas	5′ read	—
	BI160709.1	Clone IMAGE:5018991	Pancreas	5′ read	P
	BI161076.1	Clone IMAGE:5019146	Pancreas	5′ read	—
	BI161438.1	Clone IMAGE:5019572	Pancreas	5′ read	—

3	Islet cell autoantigen 1 (ICA1)				
	CB134411.1	Clone L14ChoiCK0-18-B12	Liver	5′ read	P
	BX497434.1	Clone DKFZp779M2033	Liver	5′ read	A
	BX646846.1	Clone DKFZp779C0346	Liver	5′ read	P
	AW583029.1	Clone IMAGE:5637830	Pancreas	5′ read	P
	CK904151.1	Clone IMAGE:5672417	Pancreas	5′ read	A
	BE736046.1	Clone IMAGE:3639903	Pancreas	5′ read	P
	BI715368.1	—	Pancreas	5′ read	P
	BI962895.1	Clone IMAGE:5671189	Pancreas	5′ read	—
	BI966135.1	Clone IMAGE:5672382	Pancreas	5′ read	A
	BM021952.1	Clone IMAGE:5672417	Pancreas	5′ read	A
	BU579558.1	Clone IMAGE:6121832	Pancreas	5′ read	P
	BU951015.1	Clone IMAGE:6132285	Pancreas	5′ read	P

4	SRY (sex determining region Y)-box 13 (SOX13)				
	BE563236.1	Clone IMAGE:3689361	Pancreas	5′ read	—
	BE904395.1	Clone IMAGE:3898347	Pancreas	5′ read	—
	BE905187.1	Clone IMAGE:3901107	Pancreas	5′ read	P

5	Ras-related associated with diabetes (RRAD)				
	BG250011.1	Clone IMAGE:4470428	Liver	5′ read	—
	BG250978.1	Clone IMAGE:4472119	Liver	5′ read	—
	BG252988.1	Clone IMAGE:4474056	Liver	5′ read	P
	BM967357.1	Clone IMAGE:6136533	Pancreas	5′ read	P

6	Ankyrin repeat domain 23 (ANKRD23)				
	CB159821.1	Clone L18POOL1n1-19-D04	Liver	5′ read	—
	BM127096.1	Clone IMAGE:5675155	Pancreas	5′ read	—
	BQ227733.1	Clone IMAGE:6018368	Pancreas	5′ read	—
	BU073912.1	—	Pancreas	5′ read	—

P: presence of similarity to proteins after translation and A: contains a polyadenylation signal.

**Table 4:** The ESTs and their corresponding contigs obtained from CAP3 Server.

Sl. no.	Gene name	ESTs		No. of contigs	
		Liver	Pancreas	Liver	Pancreas
1	GCK	DA640823.1	BM966889.1 BM966913.1	1	1
		DA637293.1			
		DA638310.1			

2	AVPR2	—	BI160709.1	—	1
			BI161076.1		
			BI161438.1		

3	ICA1	BX497434.1	BI715368.1	1	1
		BX646846.1	BI962895.1		

4	SOX13	—	BE563236.1	—	1
			BE904395.1		
			BE905187.1		

5	RRAD	BG250011.1	—	0	—
		BG250978.1			
		BG252988.1			

6	ANKRD23	—	BQ227733.1	—	0
			BU073912.1		

**Table 5:** BLAT output showing the alignment of contigs versus human genome sorted by score.

Query	Score	Start	End	Qsize	Identity	Chromosome	Strand
Glucokinase (GCK)							

Contig1	567	1	570	570	100.00%	7	−
Contig1	24	206	230	570	100.00%	1	−
Contig1	21	429	449	570	100.00%	4	−
Contig1	20	386	405	570	100.00%	5	+
Contig1	20	105	124	570	100.00%	3	+
Contig2	27	713	740	938	100.00%	3	−
Contig2	21	778	798	938	100.00%	1	−
Contig2	21	860	880	938	100.00%	X	+
Contig2	20	519	538	938	100.00%	4	+

Arginine vasopressin receptor 2 (AVPR2)							

Contig3	26	434	460	911	100.00%	2	−
Contig3	25	516	541	911	100.00%	2	−
Contig3	21	336	356	911	100.00%	1	−

Islet cell autoantigen 1 (ICA1)							

Contig4	586	4	593	593	100.00%	7	−
Contig4	21	570	590	593	100.00%	2	+
Contig4	20	104	123	593	100.00%	1	−
Contig4	20	105	124	593	100.00%	5	+
Contig5	963	7	992	1001	99.20%	7	−
Contig5	127	841	1001	1001	91.00%	16	+
Contig5	40	801	850	1001	90.00%	2	−
Contig5	20	545	564	1001	100.00%	1	−

Sex determining region Y-box 13 (SOX13)							

Contig6	1283	22	1340	1551	99.30%	1	+
Contig6	35	869	905	1551	97.30%	7	−
Contig6	32	869	905	1551	97.10%	6	−

**Table 6:** The InterProScan annotations for three hypothetical proteins.

Sl. no.	InterProScan	Proteins		
	applications	GCK liver	GCK pancreas	ICA1 liver
1	GENE3D	G3DSA: 3.30.420.40	G3DSA: 3.40.367.20	G3DSA: 3.20.1270.60
2	PANTHER	PTHR19443	PTHR19443	PTHR10164
3	PFAM	PF00349	PF03727	PF06456
4	PRINTS	—	PR00475	—
5	PROFILE	—	—	PS50870
6	SMART	—	—	SM01015
7	SUPER FAMILY	SSFS3067	SSFS3067	SSFS3067

## References

[1] Adams MD, Kelley JM, Gocayne JD (1991). Complementary DNA sequencing: expressed sequence tags and human genome project. *Science*.

[2] Boguski MS, Lowe TMJ, Tolstoshev CM (1993). dbEST—database for ‘expressed sequence tags’. *Nature Genetics*.

[3] Lee Y, Tsai J, Sunkara S (2005). The TIGR Gene Indices: clustering and assembling EST and know genes and integration with eukaryotic genomes. *Nucleic Acids Research*.

[4] Pontius JU, Wagner L, Schuler GD (2003). UniGene: a unified view of the transcriptome. *The NCBI Handbook*.

[5] Wheeler DL, Church DM, Federhen S (2003). Database resources of the National Center for Biotechnology. *Nucleic Acids Research*.

[6] Sehuler GD (1997). Pieces of use puzzle: expressed sequence tags and the catalog of human genes. *Journal of Molecular Medicine*.

[7] Dunstan DW, Zimmet PZ, Welborn TA (2002). The rising prevalence of diabetes and impaired glucose tolerance: the Australian diabetes, obesity and lifestyle study. *Diabetes Care*.

[8] Chakrabarti R, Misra P, Vikramadithyan RK (2004). Antidiabetic and hypolipidemic potential of DRF 2519—a dual activator of PPAR-*α* and PPAR-*γ*. *European Journal of Pharmacology*.

[9] Zhang ZY (2001). Protein tyrosine phosphatases: prospects for therapeutics. *Current Opinion in Chemical Biology*.

[10] Woodgett JR (1994). Regulation and functions of the glycogen synthase kinase-3 subfamily. *Seminars in Cancer Biology*.

[11] Woodgett JR (2001). Judging a protein by more than its name: GSK-3. *Science's STKE*.

[12] Ali A, Hoeflich KP, Woodgett JR (2001). Glycogen synthase kinase-3: properties, functions, and regulation. *Chemical Reviews*.

[13] Baker W, van den Broek A, Camon E (2000). The EMBL nucleotide sequence database. *Nucleic Acids Research*.

[14] Etzold T, Ulyanov A, Argos P (1996). SRS: information retrieval system for molecular biology data banks. *Methods in Enzymology*.

[15] Altschul SF, Madden TL, Schäffer AA (1997). Gapped BLAST and PSI-BLAST: a new generation of protein database search programs. *Nucleic Acids Research*.

[16] Shpaer EG, Robinson M, Yee D, Candlin JD, Mines R, Hunkapiller T (1996). Sensitivity and selectivity in protein similarity searches: a comparison of Smith-Waterman in hardware to BLAST and FASTA. *Genomics*.

[17] Huang X, Madan A (1999). CAP3: a DNA sequence assembly program. *Genome Research*.

[18] Lottaz C, Iseli C, Jongeneel CV, Bucher P (2003). Modeling sequencing errors by combining Hidden Markov models. *Bioinformatics*.

[19] Iseli C, Jongeneel CV, Bucher P ESTScan: a program for detecting, evaluating, and reconstructing potential coding regions in EST sequences.

[20] Zdobnov EM, Apweiler R (2001). InterProScan—an integration platform for the signature-recognition methods in InterPro. *Bioinformatics*.

[21] Hall TA (1999). BioEdit: a user-friendly biological sequence alignment editor and analysis program for Windows 95/98/NT. *Nucleic Acids Symposium Series*.

[22] Quevillon E, Silventoinen V, Pillai S (2005). InterProScan: protein domains identifier. *Nucleic Acids Research*.

[23] Nagaraj SH, Gasser RB, Ranganathan S (2006). A hitchhiker’s guide to expressed sequence tag (EST) analysis. *Briefings in Bioinformatics*.

[24] Benson DA, Karsch-Mizrachi I, Lipman DJ, Ostell J, Wheeler DL (2008). GenBank. *Nucleic Acids Research*.

[25] Bennett WS, Steitz TA (1980). Structure of a complex between yeast hexokinase A and glucose. II. Detailed comparisons of conformation and active site configuration with the native hexokinase B monomer and dimer. *Journal of Molecular Biology*.

[26] Steitz TA (1971). Structure of yeast hexokinase-B. I. Preliminary X-ray studies and subunit structure. *Journal of Molecular Biology*.

[27] Tarricone C, Xiao B, Justin N (2001). The structural basis of Arfaptin-mediated cross-talk between Rac and Arf signalling pathways. *Nature*.

[28] Spitzenberger F, Pietropaololl S, Verkade P (2003). Islet cell autoantigen of 69 kDa is an arfaptin-related protein associated with the golgi complex of insulinoma INS-1 cells. *Journal of Biological Chemistry*.

[29] Cherfils J (2001). Structural mimicry of DH domains by Arfaptin suggests a model for the recognition of Rac-GDP by its guanine nucleotide exchange factors. *FEBS Letters*.

[30] Eswar N, Marti-Renom MA, Webb B (2006). Comparative protein structure modeling using Modeller. *Current Protocols in Bioinformatics*.

[31] Marti-Renom MA, Stuart A, Fiser A, Sánchez R, Melo F, Sali A (2000). Comparative protein structure modeling of genes and genomes. *Annual Review of Biophysics and Biomolecular Structure*.

[32] Sali A, Blundell TL (1993). Comparative protein modelling by satisfaction of spatial restraints. *Journal of Molecular Biology*.

[33] Fiser A, Do RK, Sali A (2000). Modeling of loops in protein structures. *Protein Science*.

[34] Altschul SF, Gish W, Miller W, Myers EW, Lipman DJ (1990). Basic local alignment search tool. *Journal of Molecular Biology*.

[35] The Research collaborator for Structural Bioinformatics http://www.rcsb.org/pdb/home.

[36] Laskowski RA, MacArthur MW, Thornton JM, Rossmann MG, Arnold E (2001). PROCHECK: validation of protein structure coordinates. *International Tables of Crystallography, Volume F: Crystallography of Biological Macromolecules*.

[37] Miyazaki I, Gaedigk R, Hui MF (1994). Cloning of human and rat p69 cDNA, a candidate autoimmune target in type 1 diabetes. *Biochimica et Biophysica Acta*.

[38] Mally MI, Cirulli V, Hayek A, Otonkoski T (1996). ICA69 is expressed equally in the human endocrine and exocrine pancreas. *Diabetologia*.

